# *In silico* hybridization enables transcriptomic illumination of the nature and evolution of Myxozoa

**DOI:** 10.1186/s12864-015-2039-6

**Published:** 2015-10-23

**Authors:** Jonathan Foox, Maurice Ringuette, Sherwin S. Desser, Mark E. Siddall

**Affiliations:** Richard Gilder Graduate School, American Museum of Natural History, Central Park West at 79th Street, New York, NY 10024 USA; Division of Invertebrate Zoology, Sackler Institute for Comparative Genomics, American Museum of Natural History, Central Park West at 79th Street, New York, NY 10024 USA; Department of Zoology, University of Toronto, Toronto, ON M5S 3G5 Canada

**Keywords:** Myxozoa, Transcriptomes, Evolution, Host depletion, Minicollagens, Toxins

## Abstract

**Background:**

The Myxozoa, a group of oligocellular, obligate endoparasites, has long been poorly understood in an evolutionary context. Recent genome-level sequencing techniques such as RNA-seq have generated large amounts of myxozoan sequence data, providing valuable insight into their evolutionary history. However, sequences from host tissue contamination are present in next-generation sequencing reactions of myxozoan tissue, and differentiating between the two has been inadequately addressed. In order to shed light on the genetic underpinnings of myxozoan biology, assembled contigs generated from these studies that derived from the myxozoan must be decoupled from transcripts derived from host tissue and other contamination. This study describes a pipeline for categorization of transcripts asmyxozoan based on similarity searching with known host and parasite sequences, explores the extent to which host contamination is present in previously existing myxozoan datasets, and implements this pipeline on a newly sequenced transcriptome of *Myxobolus pendula*, a parasite of the common creek chub gill arch.

**Methods:**

The insilico hybridization pipeline uses iterative BLAST searching and database-driven e-value comparison to categorize transcripts as deriving from host, parasite, or other contamination. Functional genetic analysis of *M. pendula* was conducted using further BLAST searching, Hidden Markov Modeling, and sequence alignment and phylogenetic reconstruction.

**Results:**

Three RNA libraries of encysted *M. pendula* plasmodia were sequenced and subjected to the method. Nearly half of the final set of contiguous assembly sequences (47.3 %) was identified as putative myxozoan transcripts. Putative contamination was also identified in at least 1/3^rd^ of previously published myxozoan transcripts. The set of *M. pendula* transcripts was mined for a range of biologically insightful genes, including taxonomically restricted nematocyst structural proteins and nematocyst proteins identified through mass tandem spectrometry of other cnidarians. Several novel findings emerged, including a fourth myxozoan minicollagen gene, putative myxozoan toxin proteins,and extracellular matrix glycoproteins.

**Conclusions:**

This study serves as a model for the handling of next-generation myxozoan sequence. The need for careful categorization was demonstrated in both previous and new sets of myxozoan sequences. The final set of confidently assigned myxozoan transcripts can be mined for any biologically relevant gene or gene family without spurious misidentification of host contamination as a myxozoan homolog. As exemplified by *M. pendula*, the repertoire of myxozoan polar capsules may be more complex than previously thought, with an additional minicollagen homolog and putative expression of toxin proteins.

**Electronic supplementary material:**

The online version of this article (doi:10.1186/s12864-015-2039-6) contains supplementary material, which is available to authorized users.

## Background

The Myxozoa comprise microscopic, oligocellular, obligate endoparasites, with approximately 2200 species spread among over 60 genera [1]. Myxozoans mainly parasitize poikilothermic vertebrates such as fish and amphibians, as well as vermiform invertebrates such as polychaetes and oligochaetes. Myxozoans possess complex life stages with infective spores that alternate between vertebrate and invertebrate hosts. Though most myxozoans are harmless to their hosts, some are known to cause disease in commercially important wild and farmed fishes, including whirling disease in rainbow trout that can lead to nearly 90 % population loss, proliferative kidney disease in various salmonids, and intestinal giant-cystic disease in carp [2–4]. Despite global ubiquity and commercial importance, the evolutionary affinity of Myxozoa would not be settled for over a century and a half after their discovery [5]. This is owed in part to their extreme morphological degeneracy, leaving few characters to be examined comparatively (many of which contain little phylogenetic signal; see [6]), as well as resistance to culturing, marginalizing the extent to which these parasites could be studied. As a result, little was historically understood about myxozoan biology beyond localized host-parasite dynamics.

The advent of molecular systematics clarified the phylogenetic position of Myxozoa. The first inclusion of Myxozoa within a study utilizing small subunit ribosomal RNA sequence data demonstrated the metazoan origin of these parasites [7]. Soon after, additional taxon sampling and ontogenetic evidence demonstrated that myxozoans are highly derived cnidarians [8]. The evolutionary placement of Myxozoa for some time was understood only through single ribosomal gene phylogenies. The sister relationship of Myxozoa and Medosozoa, the subphylum of medusa-forming cnidarians, was revealed by the first genome-level study: 50 cloned protein-coding orthologs of the malacosporean *Buddenbrockia plumaetellae* [9]. Another study implemented phylogenetic inference of 128 protein-coding orthologs of the myxosporean *Myxobolus cerebralis* alongside *B. plumatellae* within a broad, metazoan dataset, firmly corroborating the cnidarian origin of the Myxozoa [10]. These works marked the onset of next-generation genomic study of Myxozoa in context of their nature as derived parasitic cnidarians.

At this intersection of availability of high throughput sequencing and context of cnidarian evolution, several studies in recent years have employed RNA-seq to generate transcriptomic data [11–14]. Transcriptomes are powerful tools for comparing genetic underpinnings among closely related organisms, including the exonic bases of development that are poorly understood for the Myxozoa. These initial studies have corroborated cnidarian origins by demonstrating cellular expression of taxonomically restricted genes, such as nematocyst structural minicollagens [11–14] and nematogalectins [12], a potential developmental regulatory gene [13], and proto-mesodermal genes [14].

One concern with RNA-seq approaches of non-model endoparasites is the possibility of host contamination (e.g., [15, 16]). RNA extraction is a sensitive process and total separation of parasite from host cannot be guaranteed. This concern is exacerbated by the movement away from probe-based amplification and careful cloning to generalized shotgun fragmentation sequencing techniques. Putative transcripts derived from cDNA libraries of RNA extractions of endoparasites of salmonids, a typical fish host of many myxozoan species, have been demonstrated not to have been derived from the parasite, and their misinterpretation led to incorrect inferences of biological function and evolutionary history (e.g., [17]). This is of particular concern for myxozoan research due to the histozoic nature of many species, which pervade by diffuse infiltration and thus are not easily isolated from surrounding host tissue. This includes many commercially impactful pathogens such as *Myxobolus cerebralis* and *Tetracapsuloides bryosalmonae*, the causative agents of whirling disease and proliferative kidney disease respectively in salmonids [18, 19]. Though some species proliferate through the formation of a macroscopic trophozoite in their vertebrate host that can easily be isolated, the cystic wall surrounding the trophozoite is comprised of epitheloid cells generated by the immunoresponse of the host [20], rendering inevitable the sequencing of host tissue.

Studies have implemented methods to prevent sequencing of host contamination. Careful centrifugation, pelleting, and rinsing of infective spores during RNA extraction, as well as PCR amplification using host-specific primers, has been used to ensure purity of isolation [12, 21]. Another study meticulously isolated infective stages released from oligochaetes via micropipetting [13]. However, even with such precautionary measures, studies have reported spurious findings, such as the identification of four putative myxozoan Hox homologs [22] that were later shown to be of Northern pike and bryozoan origin [9]. One solution is to generate a library in parallel of uninfected host tissue that can be used to subtract matching sequences in the infected library [12, 13]. However, this is time-consuming, costly, and may not be a realistic option for many studies. In light of the unreliability of separation of host and parasite, initial transcriptomic studies have focused only on characterization of cnidarian-specific genes, such that host contaminants were not a concern [12]. In order to facilitate wider study of myxozoan evolution with transcriptomic data cleared of contaminant transcripts, this study describes *in silico* hybridization, an iterative similarity searching and filtration pipeline. This pipeline was tested on previous datasets to explore the extent to which host transcripts pervaded. As a case study, this pipeline was then implemented on *Myxobolus pendula*, a cyst-forming parasite of gill arch of the common creek chub *Semotilus atromaculatus*. With the set of transcripts identified with reasonable certainty as exclusively myxozoan, functional genetic study can be implemented. As a framework for future transcriptomic investigation of the evolution of these highly derived parasites, this study used the filtered set of transcripts to mine for taxonomically restricted genes as well as genes with non-cnidarian homologs, describe for the first time putative venom homologs, and characterize proteins related to extracellular communication.

## Results

**Fig. 1:**
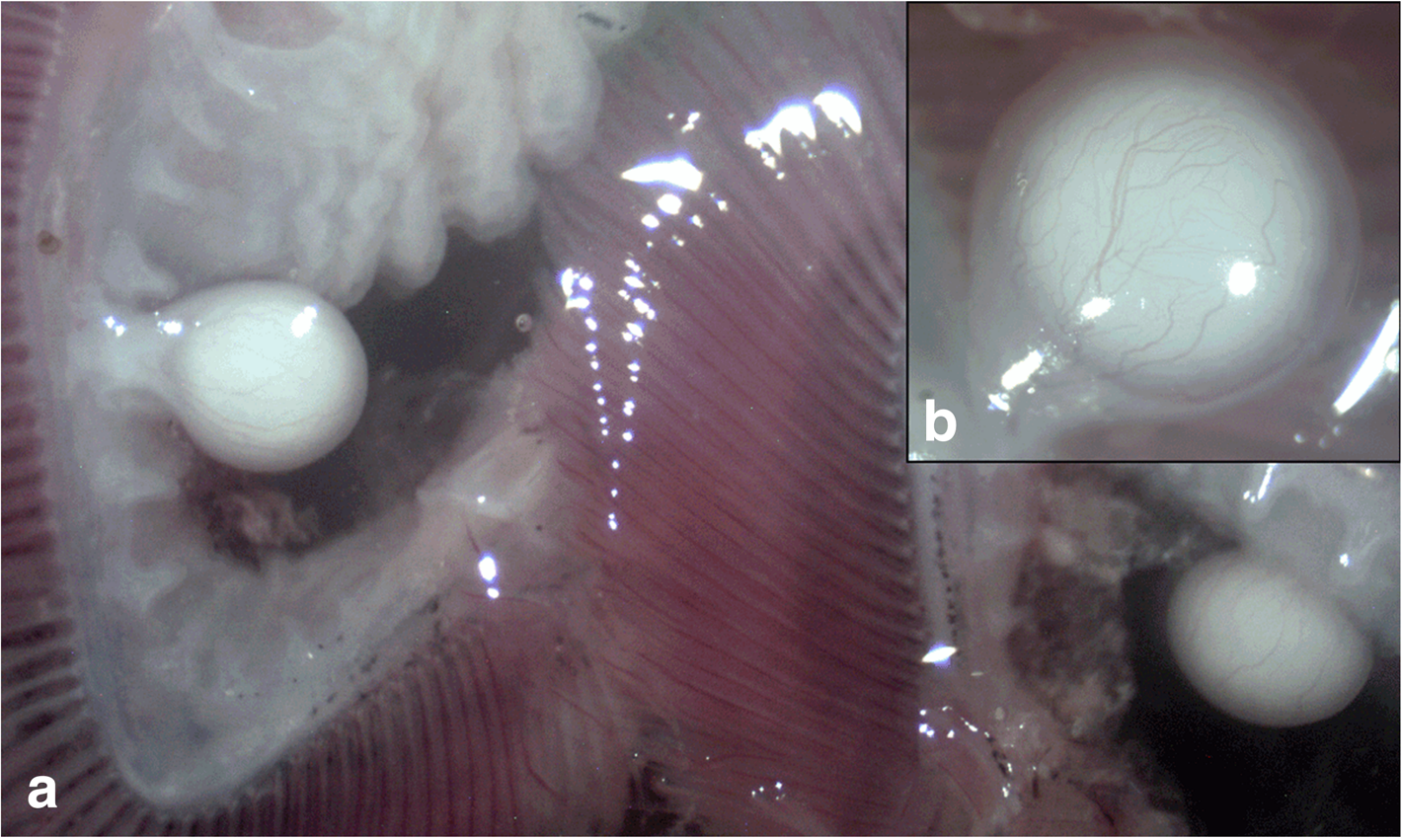
Encysted plasmodia of *Myxobolus pendula* from gill arch of *Semotilus atromaculatus*. **a** Cysts encapsulating the sporogenic stages of *M. pendula* are seen among the gill filaments. **b** Close-up of cyst revealing vascularization and attachment to gill arch by host-derived connective tissue

RNA isolated from encysted plasmodia of *Myxobolus pendula* infecting gill filaments of common creek chub *Semotilus atromaculatus* (Fig. 1) was used in a high-throughput Illumina HiSeq 2500 sequencing reaction. Three libraries of purified RNA were prepared: two from early and late stage cystic development collected summer 2004 (FCC and FLS), and a third from late stage development collected summer 2014 (MP13). These libraries occupied 1/5^th^, 1/7^th^, and 1/7^th^ of a lane of a flow cell, producing a total of 285,813,509 paired 125 bp reads. Following removal of low quality reads (Phred score ≤30, reads below 75 bp), *de novo* assembly of each library individually using the software Trinity [23], concatenation of the three sets of assembled contigs, and filtering for transcript redundancy using CD-HIT-EST [24], a final set of 575,740 transcripts was produced. Sequence and assembly statistics are summarized in Table 1.

**Table 1:** Sequencing, assembly, and *in silico* hybridization statistics

Assembly	
Raw reads	409,520,180
Reads after quality control	195,155,200
Assembled contigs	930,419
Contigs after redundancy filtration	575,740
Length min-max	200–16,127
Average length	802
N50	1288
G-C content	33.30 %
Raw reads mapped to contigs	193,101,058 (47.2 %)
*In silico* pipeline	
Only matched to Myxozoa	232,396
Stronger match to Myxozoa	39,651
Total identified as myxozoan	272,047 (47.3 %)
Only matched to host	15,057
Stronger match to host	85,043
Match to other contamination	335
Total identified as host/contamination	100,435 (17.4 %)
Too close to call^a^	14,084
No significant match found	189,174
Total without annotation	203,258 (35.3 %)

For *in silico* pipeline statistics, percentages in parentheses reflect proportion of contigs assigned to that category out of the redundancy-filtered set of contigs

^a^Matched both myxozoan and host query sets with *e*-values within two orders of magnitude of one another

**Fig. 2:**
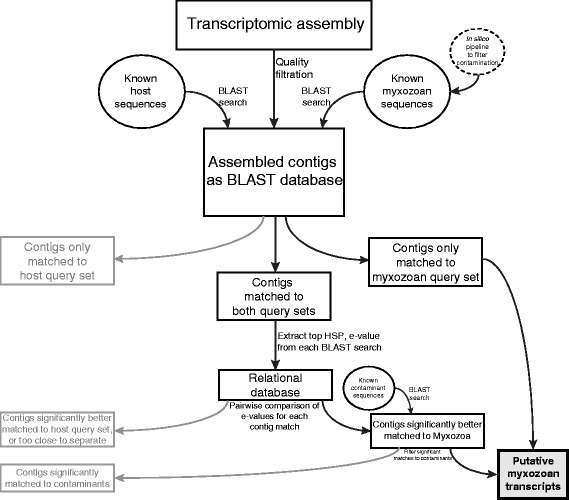
*In silico* hybridization pipeline. Diagram of the workflow used to decouple myxozoan transcripts from contaminant transcripts. Through iterative searching and comparison of *e*-values, contigs generated through transcriptomic assembly are respectively categorized

**Fig. 3:**
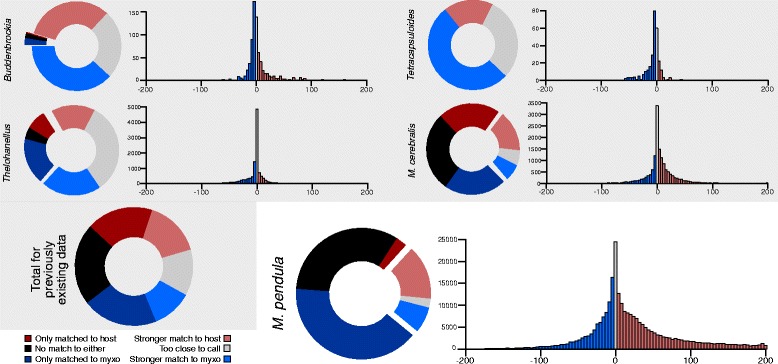
Results of *in silico* hybridization pipeline on previous data sets and on *M. pendula*. Proportional categorization of sequences from each of four previously existing myxozoan datasets, as well as a cumulative chart of all four combined, in gray box. Sequence categorization for newly generated *Myxobolus pendula* transcriptome outside of box. For each dataset, a pie chart showing breakdown of all six possible results of the *in silico* hybridization pipeline, and an associated histogram showing the resolution of “disputed” sequences that returned significant similarity against both query sets. For each histogram, the X-axis represents *e*-value ratios, in which values at either extreme are more favored for that query set, and the Y-axis represents frequency of transcripts at that *e*-value ratio

To test its efficacy, the *in silico* hybridization pipeline (Fig. 2) was implemented on four previously existing myxozoan datasets, including expressed sequence tags (ESTs) of the malacosporeans *Buddenbrockia plumatellae* [9] and *Tetracapsuloides bryosamlonae* (GenBank), predicted protein-coding peptide sequences from the genome of *Thelohanellus kitaeui* [21], and shotgun genome sequences of *Myxobolus cerebralis*, generously provided by Paulyn Cartwright at University of Kansas. Of these 70,653 myxozoan transcripts, 31.4 % (individually ranging 28.8 % - 52.1 %) were assigned as myxozoan-derived, either by matching only or better to the Myxozoa query set; 33.6 % (individually ranging 18.0 % - 36.8 %) were assigned as host-derived, either by matching only or better to fish/annelid host query sets; 35.0 % (individually ranging 29.8 % - 73.4 %) were not assigned to either, either by matching both sets within two orders of magnitude to one another or by matching neither set. The proportional breakdown of assignment for each dataset, as well as the distribution of *e*-value comparisons for transcripts that matched both query sets, are visualized in Fig. 3. Categorization of each transcript within each dataset is provided in Additional file 1.

Of the 575,740 *Myxobolus pendula* contigs, 372,482 (64.70 % of total) were definitively categorized through the *in silico* hybridization pipeline, using the aforementioned 22,193 derived sequences as the myxozoan query set. This workflow resulted in the identification of 272,047 contigs (47.25 % of total) as transcripts putatively derived from *M. pendula*, 100,435 contigs (17.44 % of total) as creek chub contaminant, and 335 contigs (0.04 % of total) as other contaminants such as gill bacteria and diplomonad parasites. By returning significant similarity to both query sets, 139,113 contigs (37.34 % of total) had to be separated by comparing *e*-values. Within this subset of “disputed” contigs, 39,986 contigs (28.74 % of disputed) returned stronger similarity to Myxozoa, 85,043 contigs (61.13 % of disputed) returned stronger similarity to the host, and 14,084 contigs (11.26 % of disputed) returned significant similarity to within two orders of magnitude to one another, thereby rendering them too close to categorize as one or the other.

**Fig. 4:**
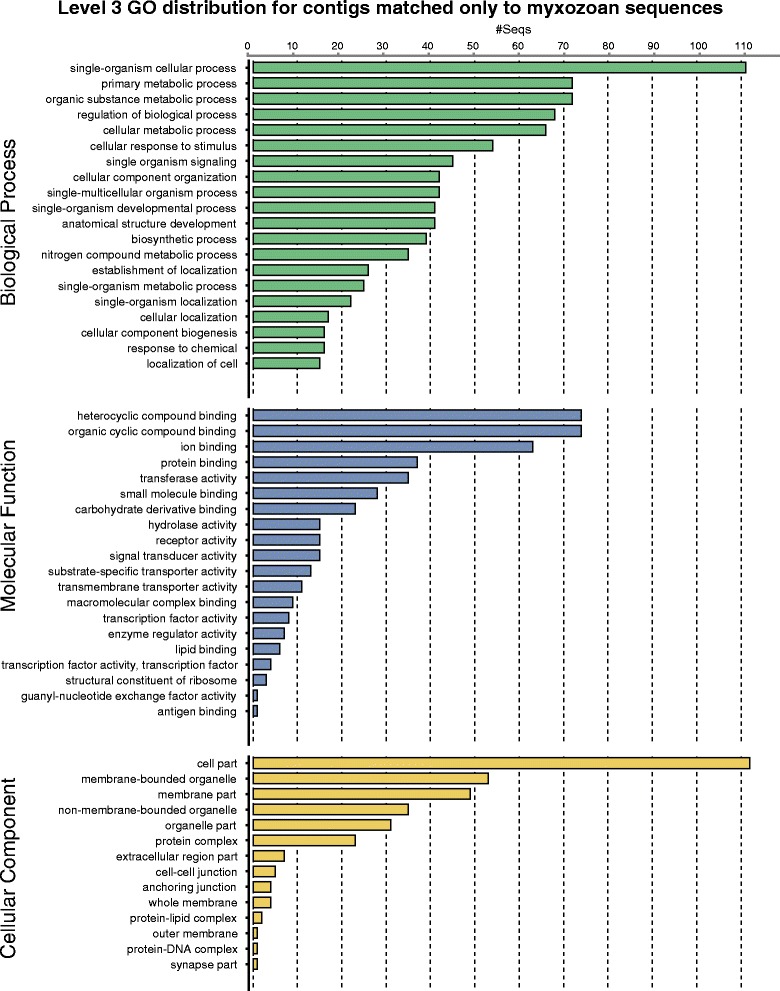
Functional annotation of transcripts matched only to Myxozoa and no other metazoan taxon. Using level 3 of Gene Ontology, functional annotation of the 2075 transcripts which were uniquely matched against myxozoan sequences and no other taxa

The remaining 203,258 contigs (35.30 % of total) were not categorized. This included the aforementioned 14,084 “too close” contigs, as well as 189,174 contigs (32.51 % of total) were found to be significantly similar to neither myxozoan nor host. As with previous datasets, proportional breakdown and distribution of *e*-value comparisons is visualized in Fig. 3. The derived set of 272,047 *M. pendula* transcripts were translated into predicted open reading frames (ORFs) using OrfPredictor [25], which generated a total of 267,995 unique peptide sequences representing 98.5 % of the derived transcript library. To test for expressional completeness with respect to the *Thelohanellus kitauei* genome [21], the predicted *T. kitaeui* proteome was searched against the set of *M. pendula* ORFs using blastp at an *e*-value of 1e^−5^, returning matches for 9208 of 16,638 (55.34 %) predicted proteins. Within the subset of predicted ORFs, 37,482 sequences (13.99 % of predicted ORFs) matched in similarity only to myxozoan data and not to any other metazoan (including cnidarian and vertebrate sequences). Functional annotational analysis, conducted using BLAST2GO [26], revealed 2075 matches (5.54 % of subset) against the nr database, whose Level 3 Gene Ontology annotational frequencies were plotted (Fig. 4).

**Fig. 5:**
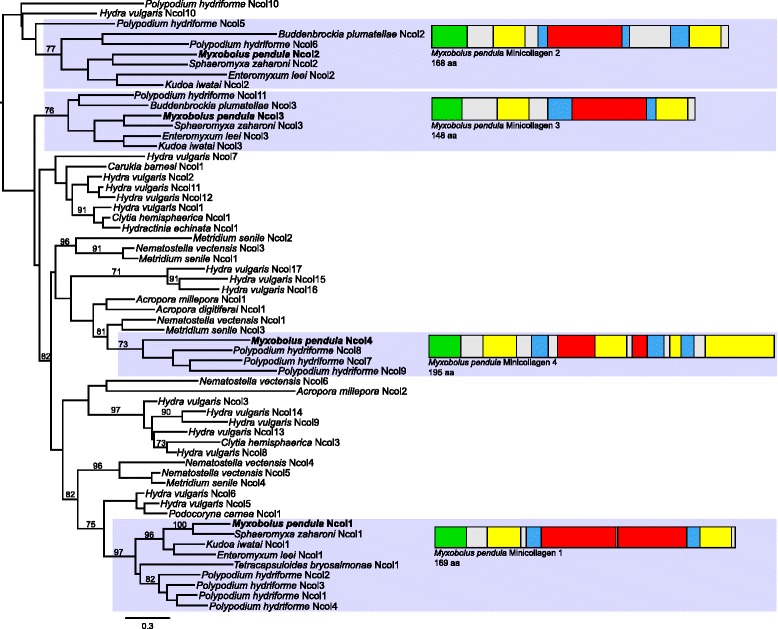
Maximum likelihood phylogenetic reconstruction of minicollagen genes across Cnidaria. Blue-shaded boxes indicate myxozoan and *Polypodium hydriforme* clades. Newly sequenced *Myxobolus pendula* minicollagens are bolded and their structures are represented by adjactent boxes, with colored boxes representing domain structure: green = signal peptide; yellow = cysteine-rich; blue = polyproline; red = polytripeptide. Values at nodes represent bootstrap supports ≥ 70. Nodes without values indicate bootstrap support below 70. AA = amino acids

**Fig. 6:**
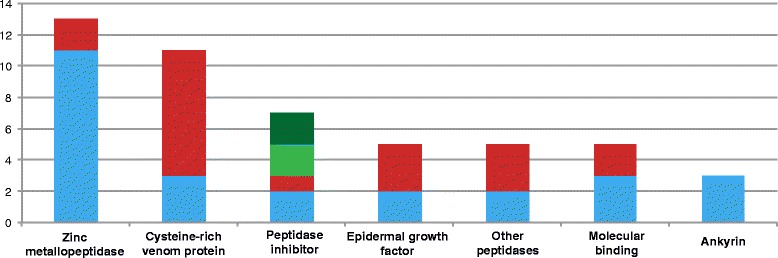
Putative *Myxobolus pendula* toxins. Matches of toxin transcripts found in the *M. pendula* transcriptome to well-annotated toxins of other cnidarians and public databases are shown divided by gene ontological categories. A total of 49 sequences were identified as candidate toxins. Blue = matched to the UniProt Animal Toxin database; red = matched to the proteome of *Hydra magnipapillata* [22]; light green = matched to *Anemonia viridis* [28]; dark green = matched to *Aurelia aurita* [28]

### Taxonomically restricted genes

Well-characterized components of the cnidarian nematocyst, such as nematogalectins, minicollagens, NOWA, and spinalin were mined for in the finalized, filtered *Myxobolus pendula* database via homologous copies from cnidarian taxa through tblastn with an *e*-value threshold of 1e^−5^. Though no putative transcripts of NOWA or spinalin were found, copies of genes within the nematogalectin and minicollagen families were discovered.

Orthologs of nematogalectin-A, nematogalectin-C, and nematogalectin-related were identified for *Myxobolus pendula*. Multiple sequence alignment of each of these putative homologs with known sequences revealed the expected domain structure for this family of genes, including a conserved signal peptide, a polytripeptide Gly-X-Y domain, and a carboxyl-terminal sugar-binding galectin domain. The gene tree phylogeny (Additional file 2) demonstrated monophyly of myxozoan orthologs of each nematogalectin gene, including a sister relationship between *M. pendula* and *Sphaeromyxa zaharoni* in each clade. No copy of nematogalectin-B was found.

Orthologs of each of the three myxozoan minicollagens identified by Shpirer et al. [12] were identified, including minicollagen I (Ncol-1), minicollagen II (Ncol-2), and minicollagen III (Ncol-3). All *M. pendula* minicollagen sequences contain the expected minicollagen domain structure, including a signal peptide, terminal cysteine-rich domains (CRDs), and a central polytripeptide Gly-X-Y domain flanked by a polyproline stretch. The central Gly-X-Y domain of *M. pendula* Ncol-1 is divided into two 14 + 13 sections of repeats by a lone alanine, rendering it a Group 1 minicollagen [27]. This domain contains only one polytripeptide stretch containing 14 tripeptides in Ncol-2 and Ncol-3, while the remaining domains are structurally identical, rendering each a Group 2 minicollagen [27]. Alignments of taxonomically restricted genes can be found in Additional file 3.

Phylogenetic analysis of all available minicollagen genes demonstrated with high bootstrap support (>70) a distinct clade for each of the three myxozoan copies (Fig. 5), with each clade spread throughout the phylogeny. Within each clade of myxozoan minicollagens, the myxosporeans are monophyletic, with a sister relationship between *Myxobolus pendula* and *Sphaeromyxa zaharoni*, as was observed in the nematogalectin family gene tree. Each clade also contains copies of *Polypodium hydriforme* minicollagens, found either between myxosporean and malacosporean minicollagens (Ncol-1 clade), sister to a malacosporean minicollagen (Ncol-2 clade), or one copy sister to a malacosporean minicollagen and another copy sister to the whole clade (Ncol-3 clade). Bayesian inference phylogenetic analyses of taxonomically restricted genes returned similar topologies to those found by maximum likelihood analysis.

An additional transcript was discovered that represents a fourth myxozoan minicollagen. This myxozoan minicollagen IV contains canonical minicollagen features, including a signal peptide, a pro-peptide region, cysteine-rich domains, and a central polytripeptide Gly-X-Y domain. Like other group 2 minicollagens, this transcript has a conserved N-terminal CRD (C…C…C…C…CC) and a unique C-terminal CRD. The minicollagen phylogeny reveals a sister relationship of this transcript with the *Polypodium hydriforme* minicollagens Ncol-7, Ncol-8, and Ncol-9. The C-terminal CRD of these four genes immediately follows the polytripeptide domain, with no polyproline stretch in between, a feature observed in no other minicollagen genes. The C-terminal CRD is interrupted by a polyproline stretch, a feature only present in *P. hydriforme* Ncol-5 through Ncol-9 and myxozoan Ncol-2*.* This putative minicollagen also contains features unique among the minicollagens. Within its polytripeptide domain, the fifth glycine residue is replaced by alanine, a similarly neutral non-polar amino acid. The polytripeptide domain contains only seven repeats, below the 12–16 range seen in other minicollagens. Three additional Gly-X-Y repeats interrupt the C-terminal CRD.

### Nematocyst proteome

The 410 unique protein sequences identified by Orbitrap MS analysis of isolated nematocysts [28] were queried against the database of *Myxobolus pendula* transcripts via tblastn with an *e*-value threshold of 1e^−5^. This search returned matches for 120 proteins. The most significantly similar *M. pendula* transcript for each matched *Hydra magnipapillata* protein was queried against the nr database in a reciprocal BLAST approach. Transcripts whose top matches were either the *Hydra* protein’s annotation or an “uncharacterized/predicted” protein within Cnidaria were retained, resulting in 82 *M. pendula* proteins that match the proteome of the *Hydra* nematocyst (Additional file 4).

Of the 82 proteins identified, 15 matched *Hydra* extracellular matrix (ECM) glycoproteins. Three prominent ECM domains were repeatedly identified, including thrombospondin type-1, von Willebrand factor-A, and Laminin-G (Additional file 5). Other prevalent ECM domains encoded by the contigs were found to have homology to members of the SEA, chitin-binding, and discoidin superfamilies.

As a follow-up to the 14 venom proteins matched between *Hydra* and *M. pendula*, a more detailed analysis of toxin proteins was conducted with a wider set of query sequences (see Methods). A total of 49 sequences were identified as candidate toxins (Additional file 6). These transcripts spanned several toxin classes included metallopeptidases, other peptidases, peptidase inhibitors, and cysteine-rich secreted proteins, as well as toxin-associated classes such as molecular binding proteins (Fig. 6). Despite the significantly larger number of non-cnidarian query sequences from the animal toxin database (6214) compared to cnidarian sequences from recent MS/MS studies and within the UniProt database (288), the number of matches was nearly even, with 26 identifications deriving from the UniProt database and 23 from cnidarian queries. Of the 49 toxin candidates, 16 were predicted to code for signal peptides using SignalP 4.0 [29]. Of the 33 sequences without a signal peptide, 17 were found to be incomplete at their N-terminus, indicating that upward of 33 of 49 *M. pendula* toxin candidates code for signal peptides.

## Discussion

In an effort to decouple parasite transcripts from host contaminants, an *in silico* hybridization pipeline was developed (Fig. 2). Through six possible outcomes, transcripts were categorized as: derived from the myxozoan, either by (1) only returning significant similarity to the myxozoan query set or (2) having a myxozoan similarity score more than two orders of magnitude greater than the host similarity score; derived from the host, either by (3) only returning significant similarity to the host query set or (4) having a host similarity score more than two orders of magnitude greater than the myxozoan similarity score; no categorization, either because (5) similarity was detected in both and the respective similarity scores were too close to distinguish or (6) no significant similarity was detected in either BLAST search. The utility of this method was tested on previously existing myxozoan transcriptomic data as well as the newly generated transcriptome of *Myxobolus pendula* (Fig. 3). The proportional distribution of these six assignment outcomes, represented as pie charts, was variable among datasets (see Additional file 1 for raw numerical and percentage breakdowns). For *M. cerebralis*, the majority of transcripts were matched to either only one or neither query set; for *Tetracapsuloides*, all transcripts were matched to both query sets. Although both *Buddenbrockia* and *Thelohanellus* also had a majority of transcripts match both query sets, the former returned a greater resolution of sequences as myxozoan, whereas the latter returned many more sequences that were too close in similarity to distinguish. *M. pendula* showed a similar distribution to its congener *M. cerebralis*, in which the majority of transcripts matched one or neither query set, and the majority of disputed contigs were assigned to the host.

The histograms associated with each pie chart, representing the resolution of “disputed” contigs matched by both datasets, show the extent to which *e*-values were favored by either query. For each taxon, the spread of preferences roughly resembles a normal distribution, with few cases at either extreme, more towards the center, and the most (or second most) frequent in that central “too close to call” range. In particular, the proportion of ambiguity in the *Thelohanellus* data (of disputed contigs, 46.86 % too close versus 53.14 % resolved either way) underscores one limitation of the method, which can be assuaged by improved query sets (see below) or by secondary analyses such as domain structure. The largest of these datasets, *M. pendula* had many more contigs resolved as host at higher *e*-value differentials, much more so than did its close relative *M. cerebralis*. This indicates that as datasets grow, the level of obvious host contamination will grow too. Perhaps most tellingly, the cumulative distribution for previous datasets was roughly 1/3^rd^ of reads matching Myxozoa, 1/3^rd^ matching *Danio rerio* for myxosporean data or *Capitella teleta* for malacosporean data, and 1/3^rd^ unannotated. Under the most generous interpretation of these results, in which all transcripts that were “too close” and all transcripts that didn’t match either query set are assumed to be myxozoan-derived, a third of the data still putatively derived from the host. This demonstrates a significant degree of contamination and the need for examination thereof within this system.

Using the filtered datasets as the myxozoan query set, this pipeline was implemented on RNA sequenced from encysted plasmodia of *Myxobolus pendula* (Fig. 1). Approximately half of the assembled contigs were identified with reasonable confidence as *Myxobolus pendula* transcripts (Fig. 3). As the database of myxozoan genetic data grows, more of these sequences can be incorporated into the myxozoan query set within the pipeline, and this increased set of sequences will likely enable more contigs to be identified as myxozoan with greater confidence. Nearly 1/5^th^ of the transcripts were identified as deriving from the creek chub host, and a few hundred more as other contamination, such as gill bacteria or flagellates. It will be similarly important to have genomic data available for hosts and potential microbial contaminants in order to filter non-myxozoan transcripts. Fortunately, because so many intermediate vertebrate hosts of myxozoans are closely related to model organisms (e.g., *Danio rerio* or species of *Xenopus*), the availability of such data is of little concern. Of the remaining sequences, over 1/3^rd^ could not be annotated as deriving either from *M. pendula* or from creek chub; as a corollary to the foregoing, this number will be reduced as query sets are augmented.

Within the final set of *M. pendula* transcripts, 37,482 were matched only to myxozoan query sequences and not to any other metazoan. This set of transcripts seemingly restricted to the Myxozoa represents an interesting baseline for future study of specialization of these animals towards morphologically degenerate parasitism. These sequences are available as a FASTA file in Additional file 7. When subjected to functional annotation using BLAST2GO [26] at an *e*-value cutoff of 1e^−5^, only 2075 transcripts (5.56 %) within this subset returned annotational matches. The overwhelming lack of similarity may be reflective of the highly divergent genetic nature of Myxozoa, as has observed in known myxozoan ribosomal markers (e.g., [30]) and in the mitochondrial genome [31]. The scarcity of matches also may be compounded by the lack of currently available myxozoan genetic data in the nr database. The Gene Ontology terms for those with a match were visualized categorically at Level 3 of gene ontology (Fig. 4). In all three classifications, clusters relating to cellular activity (e.g., cellular process, metabolic process, cyclic binding, signaling, etc.) were much more highly represented than clusters relating to development and reproduction (e.g., growth, structure sizing). This result was expected given that the collected plasmodia were not believed to be in stages of development. In addition to a general increase in publicly available genetic data, sequences generated from different stages of development will facilitate a more complete understanding of lineage-specific myxozoan gene families.

One consideration with this approach is that identifiable contigs will be limited to protein-coding genes. Despite their apparent non-protein-coding nature, a significant number of expressed transcripts within invertebrates are known to have regulatory and yet unknown functions. Such transcripts cannot be identified by a comparative pipeline that is built on similarity searching with known protein-coding mRNA sequences. In order to study transcripts such as long non-coding RNAs (lncRNAs), future genomic studies will have to incorporate additional measures to identify and characterize them. This will further reduce the number of unannotated sequences, as these transcripts may in part be comprised of genes such as lncRNAs as well as protein-coding genes yet unidentifiable with current query sets.

In light of the difficulty of total expulsion of host tissue during nucleic acid extraction, this method offers (at least with respect to protein-coding genes) an *a posteriori* bioinformatic solution to ensure proper identification and filtration of contaminant sequences. To the extent that is possible when working with non-model microscopic endoparasites and currently available public data, this approach eliminates spurious identification of contaminant transcripts as being parasite-derived, such as the identification of bryozoan Hox genes as myxozoan [9], and thereby enables functional genetic searching beyond taxonomically restricted genes. Because this method compares search results among query sets, it is a more thorough and precise approach than a one step depletion of contaminants from a transcriptomic library by similarity searching against a set of host sequences at some similarity threshold. This single step removal of contigs that match non-parasite at some *e*-value may exclude ancient, highly conserved genes that return high similarity against both parasite and non-parasite. Obversely, this may incorrectly retain genes that are dissimilar to non-parasite but are even more dissimilar to the parasite.

The conversion of the transcriptomic library of interest into a database against which known sequences are queried is critical in order to ensure comparability of similarity scores. *E*-values, the calculation of which is dependent upon the size of the database, can only be compared across searches against a common database. Use of unannotated transcripts as queries against variably sized databases of known sequences would violate this requisite for comparison of similarity score. Sorting similarity search output by subject and *e*-value returns the most significantly similar and therefore most likely identification of each contig within each search, and these identifications can be compared across searches.

### Functional genetics

As demonstrated for several myxozoan species in a recent study of polar capsule structural proteins [12], *Myxobolus pendula* possesses three copies of the nematogalectin family, including nematogalectin A, nemgalectin C, and nemgalectin-related. The lack of nematogalectin B, the exons of which exist within the same gene as nematogalectin A and are expressed by alternative splicing, is unsurprising due to its ontogenetic relation to the development of barbs and spines of the proximal tubule of *Hydra* [32], features which are not present in myxozoan polar filaments. Secondary loss of nematogalectin B could in part have shaped the relative simplicity of the myxozoan polar filament repertoire. Similarly, the absence of spinalin and NOWA, the other two well-characterized nematocyst proteins, is unsurprising given their lack of homologs outside of *Hydra* [33]. Phylogenetic reconstruction of the nemgal family, in which *M. pendula* is monophyletic with other myxozoans (Additional file 2), reveals a basal relationship of myxozoans with other cnidarians in all three nematogalectin genes that myxozoans possess. This is reflective of an ancient divergence of this critical component of the nematocyst tubule.

Neither minicollagen group type nor copy number exhibited strong signal in the phylogenetic reconstruction of this family. This, in combination with poor bootstrap support for most internal nodes, reflects the rapid evolution of these short proteins. Despite this, minicollagens of *M. pendula* and other myxosporean species were monophyletic for all gene copies, reinforcing the phylogenetic utility of this gene family. In each minicollagen gene clade, myxozoan taxa exhibited sister relationships with *Polypodium hydriforme*, a parasite of acipenseriform oocytes. Like myxozoans, *P. hydriforme* is an obligate endoparasite, develops via “cell-within-a-cell” ontogeny, possesses atrichous isorhizan nematocysts, and is believed to be a highly modified, morphologically degenerate cnidarian. A sister relationship between myxozoans and *P. hydriforme* has been demonstrated in numerous studies (e.g., [13, 26, 34]), and the clade comprised of these two taxa has been labeled “Endocnidozoa” (albeit in an effort to label this grouping at the base of the Bilateria rather than derived within Cnidaria) [35]. The phylogenetic findings within this study contribute to the growing body of evidence evolutionarily uniting these parasites.

Beyond the three previously described myxozoan minicollagen genes [12], a fourth copy was discovered in the *M. pendula* transcriptome. Though the diversity of myxozoan minicollagens (4) is still much lower than the putative sister taxon *Polypodium hydriforme* (11) or free-living cnidarians such as *Hydra magnipaillata* (21), this finding marks the fourth time that the described diversity of polar filament minicollagens has been augmented [11, 12, 14], suggesting greater polar filament complexity than previously imagined. The adjoined polytripeptide and cysteine-rich domains of myxozoan minicollagen IV and *P. hydriforme* Ncol7-9, found in no other minicollagen copies, is strong evidence of the homology of these genes. This relationship is corroborated by monophyly of these genes in the phylogenetic reconstruction of the minicollagen family (Fig. 5). The fifth glycine residue within the polytripeptide domain of myxozoan minicollagen IV is replaced by alanine, a feature observed in no other minicollagen. The substitution of glycine with alanine in the central core of helical domains, such as the triple helix-forming polytripeptide within minicollagens, has been shown to stabilize the protein [36]. This primary structure of minicollagens is therefore more malleable than previously thought, a notable observation given that these proteins that are known for their considerable tensile stability as major components of one of the most osmotically pressurized cellular products known [37].

In addition to structural collagen and proteoglycan proteins, several glycoprotein extracellular matrix molecules were identified in the *M. pendula* transcriptome. For their role in determining shape and physical properties of tissues as well as regulating all cellular activities, ECM molecules have played an indispensable role in the development and evolution of multicellular organisms with species-specific innovations. This renders ECM molecules important for comparative study of the biology of the highly degenerate Myxozoa among free-living cnidarian relatives. Three glycoprotein domains were found prominently in *M. pendula*, including laminin-G, thrombospondin type-1, and von Willebrand factor-A (Additional file 5). The Lam-G domain repeats of the C-terminal α-subunit laminin trimers bind to integrins, heparin sulfate proteoglycans and α-dystroglycan [38], promoting the polarization of epithelial cells. Thrombospondin TSP-1 domains, which are found in many ECM glycoproteins, induce signal transduction by binding to several cytokines and growth factors [39]. vWA domains are involved in regulating a wide variety of cellular functions are found within integrin subunits, collagens and several other ECM glycoproteins [40]. However, it remains to be determined if putatively homologous domains expressed by *M. pendula* have equivalent functions. Identification or assignment of biological functions to ECM molecules is challenging for many reasons. The functions of ECM glycoproteins are modulated by posttranslational modifications, such as glycosylation, phosphorylation and sulfation. Alternative splicing can also generate multiple isoforms. The genomes of *M. pendula* and other cnidarians code for a variety of matrix remodeling metalloproteinases and other peptidases that could cleave and alter the activity of specific ECM proteins at different stages of sporogenesis. Moreover, ECM glycoproteins cannot be identified by the presence of a specific domain, since domains are shared by multiple ECM glycoproteins; rather it is the specific combination and the order of domains that serve as a more reliable diagnostic tool. Hence, further bioinformatic analysis of the contigs generated from this study is required to gain a better overview of the complexity of the *M. pendula* secretome and the phylogenetic relationship of individual glycoproteins to known and uncharacterized ECM glycoproteins.

On the basis of amino acid sequence and domain similarity to toxins described from cnidarians (*Hydra magnipapillata* [28]*, Anemonia viridis* and *Aurelia aurita* [41]) and the UniProt animal toxin database, a set of 49 transcripts was identified as putative myxozoan toxins. These candidate sequences represent a similar diversity of envenomation-related proteins as found in other cnidarians, including peptidases, cytolysins, and molecular binders. This is the first recorded evidence of toxin proteins expressed within myxozoans. The expression of toxin proteins within polar capsules contravenes previous assumptions about myxozoan biology. Polar filaments have been described as injecting neither venoms nor digestive enzymes, but rather acting as anchors to the next host in the myxozoan life cycle [42]. More specifically, polar capsules have been shown ontogenetically to be homologous to the atrichous isorhiza [8], a type of nematocyst known within Cnidaria as exclusively producing sticky secretions that assist in either capture or adhesion to integument of prey [43]. The everted tubule of a polar capsule is holotrichous and spineless, and has been shown to enable attachment to host by infective myxozoan spores loose in the water column [44]. These findings suggest that polar filaments are more functionally complex than previously imagined, and may possibly utilize envenomation during host infiltration. A recent study of the venom proteome of the box jelly *Chironex fleckeri* [45] has concomitantly raised doubt as to the nature of this nematocyst type shared by myxozoans, demonstrating little difference in the toxin contents of isorhizas and mastigophores, which are known to contain the lethal components of cubozoan venoms [46].

Nevertheless, sequence similarity and even domain conservation cannot confirm the expression of toxins within the myxozoan polar capsule. Despite apparent homology, venom proteins have been shown to adapt to roles unrelated to envenomation, such as metallopeptidases in *Hydractinia echinata* participating in cellular development [47]. A recent study producing the genome of *Thelohanellus kitauei* has described peptidases and peptidase inhibitors similar to those found presently in *Myxobolus pendula* as functioning in nutrient absorption [21]. That nearly half of the identified homologs matched proteins identified through tandem mass spectrometry of isolated nematocysts lends credence to the polar capsular origin of these putative toxins. A mass spectrometry analysis of sequences derived from isolated myxozoan polar capsule proteins cross-referenced with the candidates identified from the *M. pendula* transcriptome is necessary to define the nature of these transcripts.

## Conclusion

The advent of next generation sequencing of myxozoans in combination with strong evidence for their cnidarian origins has set the stage for genome-level myxozoan research. The pipeline described herein enables confident decoupling of myxozoan transcripts from contaminant transcripts generated during a sequencing reaction. The implementation of this *in silico* hybridization pipeline on previously existing myxozoan datasets reveals varying proportions of putative contamination and demonstrates the need for careful, rigorous examination of data generated from these microscopic parasites.

Implementation of this workflow on the creek chub parasite *Myxobolus pendula* serves as a model for exploring the genetic underpinnings of myxozoan biology and evolution, including taxonomically restricted structural genes, proteomic profile comparisons to other cnidarians, extracellular matrix motif proteins, and potentially pathogenically important toxin genes. Expressional studies can be used to explore numerous other biological avenues, such as the evolution of germ layers, cellular communication, or host recognition pathways. In any such targeted study, proper identification of myxozoan transcripts is paramount, and can be achieved using this *in silico* hybridization method.

## Methods

### Sampling and sequencing

Individuals of the common creek chub *Semotilus atromaculatus* were caught using Gee® minnow traps baited with Purina puppy chow® in Lake Sasajewun (45°35′30″ N, 78°31′30″ W), Algonquin Park, Ontario in summer 2004 and summer 2014. All necessary permits were acquired from the Canadian Ministry of Natural Resources. Gill filaments of pithed fish were isolated and inspected under a stereomicroscope, and encysted plasmodia of *Myxobolus pendula* were excised by incision through peduncle connected to host gill arch, with care to minimize adjacent host tissue captured. Spores of *M. pendula* were confirmed by examination of punctured plasmodia under light microscopy. Excised plasmodia were immediately fixed in RNA*later* (QIAGEN) and stored initially at −20 °C, then transferred to −80 °C for long-term storage.

RNA was isolated using a hybrid novel/RNEasy Mini Kit (QIAGEN) protocol. Thawed samples, each containing 2–6 excised cysts, were centrifuged for five minutes at 12,000 rpm to pellet plasmodia and loose myxospores. RNA*later* was removed and replaced with 20 μl RNase-free distilled water and samples were refrozen at −20 °C for ten minutes. Two rounds of 250 μl of TRIzol® Reagent was added, with vortexing and maceration with a pestle of the samples in between. Samples were passed through a syringe five times, 100 μl of chloroform was added, and samples were cold centrifuged at 11,000 rpm for 15 min. The clear aqueous phase was transferred to a new tube with equal volume of 70 % ethanol, at which point the standard protocol of the RNEasy Mini Kit was followed starting at step four. Aliquots of final RNA isolations were quantified using an Agilent RNA 6000 Nano Kit on an Agilent 2100 Bioanalyzer. RNA stocks were immediately stored at −80 °C. The remaining DNA interphase was separately collected and isolated using the standard protocol of the DNEasy Blood and Tissue Kit (QIAGEN). Using standard eukaryotic primers ERIB1 5′-ACCTGGTTGATCCTGCCAG-3′ and ERIB10 5′-CTTCCGCAGGTTCACCTACGG-3′, the 18S rRNA gene was amplified and sequenced to confirm the isolated myxozoan species.

RNA isolates with an RNA Integrity Number (RIN) above 7.0 were sent for polyadenylation selected RNA library preparation with a proprietary kit and high throughput sequencing at the New York Genome Center. A total of three *Myxobolus pendula* isolates were sent (labeled “FCC”, “FLS”, and “MP13”), each receiving 1/5^th^, 1/5^th^, and 1/7^th^ of a lane respectively in a multiplexed sequencing reaction on an Illumina HiSeq 2500 utilizing paired-end 125 bp reads. The resulting sequences were trimmed using the fastx toolkit (http://hannonlab.cshl.edu/fastx_toolkit/), resulting in the removal of key/adaptor sequences, poly-A tails, and low quality reads (Phred score ≤30). The quality of remaining sequences was verified using FastQC (http://www.bioinformatics.babraham.ac.uk/projects/fastqc). Filtered reads of each library were independently assembled using default features of Trinity *de novo* assembler version r20130225 [23]. Raw reads were mapped back to assembled contigs using BWA [48]. The reads generated by the sequencing reaction were deposited to the NCBI Short Read Archive under accession SRS1077053 with BioProject PRJNA296504. All data handling and subsequent analysis was performed on the Malthus computer cluster at the American Museum of Natural History.

### In silico *hybridization*

A novel pipeline was constructed in order to separate contigs representative of *Myxobolus pendula* transcripts from host contaminant and chimeric contigs in each of the transcriptomic assemblies by “hybridizing” (matching with significant similarity) them to known sequences. The following was applied to each assembly independently. To search for parasite transcripts, all available myxozoan sequences were downloaded from the National Center for Biotechnology Information (NCBI) GenBank, including expressed sequence tags (ESTs) of the malacosporeans *Buddenbrockia plumatellae* and *Tetracapsuloides bryosalmonae*, and predicted protein-coding peptide sequences from the genome of *Thelohanellus kitaeui* [21]. Shotgun genome sequences of *Myxobolus cerebralis* were also included, generously provided by Paulyn Cartwright at University of Kansas. These sequences were subjected to this pipeline (see results) and the filtered dataset was used as the myxozoan query set (see below). In addition, because of the unusually high rate of myxozoan sequence evolution combined with the paucity of available myxozoan sequences in public databases, ESTs and predicted peptide sequences were downloaded for representatives of major groups within Cnidaria, the phylum in which Myxozoa belongs. The taxa chosen included the anthozoans *Aiptasia pallida*, *Anemonia viridis*, and *Metridium senile*, the hydrozoans *Clytia hemisphaerica* and *Hydractinia echinata*, and the scyphozoan *Aurelia aurita*. Collectively, the myxozoan and cnidarian set of sequences comprised the myxozoan query set. To search for host contaminant transcripts, protein-coding peptide sequences predicted by the Ensembl genebuild pipeline from the *D. rerio* genome were downloaded. The cyprinid *D. rerio* represents the closest relative of *Semotilus atromaculatus* with a well-annotated genome. This set of sequences was used as the host query set.

Rather than use assembly contigs as queries against databases of the myxozoan and host sets, a database of the assembled contigs was generated, against which all BLAST queries were implemented. This was done because the metric chosen to compare BLAST outputs was *e*-value, a measure of probability of matching a query to its target by chance (essentially calculating the likelihood of a false positive) whose calculation is dependent upon the size of the database being queried. Therefore, querying a consistently sized database is critical for *e*-value comparisons across searches.

For searches using peptide sequences as a query, the tblastn program was used. For searches using nucleotide sequences as a query, the tblastx program was used. In each search, the maximum number of high-scoring segment pairs (HSPs) (−max_hsps_per_subject) was set to 1, but the maximum number of target sequences (−max_target_seqs) was not. This ensured that the BLAST output only reported the most significantly similar match between each contig and the query that matched it, but allowed multiple contigs to be matched by the same known query. The *e*-value threshold was set to 2, in order to capture sequences with low complexity that would be locally insignificant under a more stringent *e*-value setting, and to maximize the number of contigs identified.

For each contig matched within the myxozoan and host BLAST searches, custom Python scripts were created to extract the most significant high-scoring segment pair (HSP) as well as its corresponding *e*-value from each BLAST output. Contigs that were matched only by the parasite or host sets were categorized as such respectively. Contigs that were matched in both searches were identified using the web tool Venny 2.0 (http://bioinfogp.cnb.csic.es/tools/venny/). These “disputed” contigs and their corresponding *e*-values from each search were imported into a relational database using MySQL, whereupon the *e*-values from the host and parasite searches generated for each contig could be pairwise compared. Any contig whose myxozoan *e*-value was at least three orders of magnitude smaller than its corresponding host *e*-value was selected and appended to the set that matched only to Myxozoa.

Finally, this set of putative myxozoan transcripts was filtered against predicted peptide sequences from genomes of potential contaminants, including common gill bacteria such as *Cytophaga hutchinsonii, Flavobacterium branchiophilum*, *Flexibacter litoralis*, *Nocardia brasiliensis*, and *Yersinia enterocolitica,* the parasitic diplomonads *Giardia lamblia* and *Hexamita inflata*, the ciliate causative agent of marine ich *Ichthyophthirius multifilis*, and *Homo sapiens*. This step was done as late as possible due to the size of this query set, which requires considerable computational time. The final sets of parasite transcripts from each of the three transcriptomic libraries were labeled with a library-specific prefix and the sequences were concatenated as the final myxozoan dataset.

### Transcriptomic mining

The final library of putative *Myxobolus pendula* transcripts was converted into a BLAST database against which genes of interest were searched. Peptide sequences of well-characterized genes restricted to the phylum Cnidaria (“Taxonomically Restricted Genes”) were downloaded from the NCBI nr database. Additional sequences were retrieved from the supplementary material of Shpirer et al. [12]. This included 57 minicollagen sequences, 47 nematogalectin sequences, and one sequence for each of Nematocyst Outer Wall Antigen (NOWA) and spinalin. A tblastn search using an *e*-value threshold of 1e^−5^ was implemented for each protein family. Transcripts that were identified as potential homologs were translated into amino acid sequences, and open reading frames were queried against the nr database using blastp in a reciprocal BLAST search approach. Any sequences that reciprocally matched their protein family were retained and identical transcripts were pruned.

This same approach was used to identify proteins shared with the *Hydra magnipapillata* proteome [28]. The 410 nematocyst proteins identified by Balasubramanian et al. [28] were queried against the *M. pendula* database using tblastn with an *e*-value cutoff of 1e^−5^. For each matched query, the contig that matched most significantly was translated into an amino acid sequence and queried reciprocally against nr using blastp. Sequences with top hits either matching the protein family of the *Hydra* query or matching an “uncharacterized/predicted protein” but within the phylum Cnidaria were retained.

As a follow-up analysis to explore putative toxin homologs, toxin genes that were verified as expressed within nematocysts through tandem mass spectrometry and were available in public databases were used as queries. As additional data, the complete set of 6381 verified toxin proteins from the June 1, 2015 release of the UniProt animal toxin database [49] were used as queries. In order to prevent misidentification based on matching of a single toxin-like domain, only hits that spanned ≥75 % of the length of the transcript were retained. Candidates were reciprocally BLAST searched to ensure protein family identification. As a measure beyond amino acid similarity searching, domain structures of candidate transcripts were tested for functional similarity, inferred based on conserved domain motifs identified through InterProScan 5.0 [50] under default parameters.

For each gene family of interest above (e.g., minicollagens, toxins, etc.), in addition to BLAST searching, multiple sequence alignments (see below) were converted into Hidden Markov Model (HMM) profiles and used in HMMer 3.1b2 [51] to identify sequences that were missed by BLAST searching. For each match, HMMer reports an *e*-value for the global alignment, an *e*-value for the single best domain score, and a “bias” estimate to detect artificial significant global similarity achieved through repeated matching of weakly-scoring domains. This is common when searching for proteins with low sequence complexity, including structural proteins such as minicollagens and nematogalectins. To account for this, only transcripts for which the global *e*-value was no more than 5 orders of magnitude greater than the single best domain *e*-value were retained. These additional gene candidates were also reciprocal BLAST queried to ensure family matching.

Multiple sequence alignments of candidate transcripts and their respective homologs were generated using the E-INS-i algorithm in MAFFT v7.0 [52]. Poorly conserved columns were masked using Gblocks0.91b [53] with a minimum of 50 % of sequences for a conserved position, 75 % of sequences for a flank position, 10 minimum length of a conserved block, eight maximum contiguous nonconserved positions, and allowing 50 % of taxa with gaps per site. For phylogenetic analysis, protein substitution models were selected using ProtTest v3.1 [54], with Bossum62 + I + G + F chosen for the minicollagen and nematogalectin alignments. Maximum likelihood analyses were conducted through PhyML [55] using the models identified by ProtTest, with 10 random starting trees, NNI and SPR branch swapping, and 100 bootstrap pseudoreplicates to calculate support. Bayesian analyses were conducted using MrBayes 3.2 [56] under the models identified by ProtTest, with two runs with four chains each, sampling every 1000 generation, 25 % burn-in, and termination when the standard deviation of split frequencies reached below 0.01.

## Additional files

Additional file 1:
**Statistics and sequence labeling of existing datasets.** For each existing myxozoan dataset on which the *in silico* pipeline was implemented, percent breakdown and categorization of each sequence, as well as cumulative statistics. (XLSX 286 kb) Additional file 2:
**Maximum likelihood phylogenetic reconstruction of nematogalectin family genes.** Newly sequenced *Myxobolus pendula* nematogalectins are bolded. Numbers at nodes represent bootstrap support values. Values at nodes represent bootstrap supports ≥ 70. Nodes without values indicate bootstrap support below 70. (JPEG 175 kb) Additional file 3:
**Alignments of taxonomically restricted genes, including minicollagens (4) and nematogalectins (3).** Taxa include previously sequenced myxozoans, newly sequenced *Myxobolus pendula*, and *Polypodium hydriforme.* Colored boxes refer to domain structure. Green = signal peptide; gray = propeptide; yellow = cysteine-rich; blue = polyproline (minicollagens), sugar-binding galectin (nematogalectins); red = polytripeptide; orange = tripeptide with alanine replacing glycine. Cysteine residues within cysteine-rich domains are shaded red. (DOCX 234 kb) Additional file 4:
**Matches of** ***Myxobolus pendula*** **transcripts to** ***Hydra*** **proteomic sequences.** Rows in which *M. pendula* transcripts matched significantly to *Hydra magnipapillata* proteomic sequences [22] are highlighted, and the top match using BLASTp against nr is indicated. A total of 82 out of 410 *Hydra* sequences returned significant matches. (XLSX 63 kb) Additional file 5:
**Extracellular Matrix glycoproteins of** ***Myxobolus pendula.*** The 15 transcripts that matched to *Hydra* extracellular matrix proteins were analyzed for domain structure and BLASTp matching to nr. Three of 15 transcripts were observed to contain signal peptides. (XLSX 40 kb) Additional file 6:
**Ontological categorization of 49 putative toxin transcripts.** The *M. pendula* transcripts that were shown to have homology to venoms found in *Hydra*, other cnidarians, and/or the UniProt animal toxin database are shown. This includes function of matched toxin, domain structural analysis, and gene ontology (biological process, molecular function, cellular component). Sixteen of 49 toxins were observed to contain signal peptides. (XLSX 34 kb) Additional file 7:
**Transcripts matched only to Myxozoa.** The 37,482 transcripts of *Myxobolus pendula* that were matched only to myxozoan query sequences and to no other taxon. (FA 22455 kb)
