# Supplementary figures and images for: In silico hybridization enables transcriptomic illumination of the nature and evolution of Myxozoa
Source: BMC Genomics. 2015 Oct 23;16:840. doi: 10.1186/s12864-015-2039-6 (PMC4619090; doi:10.1186/s12864-015-2039-6)

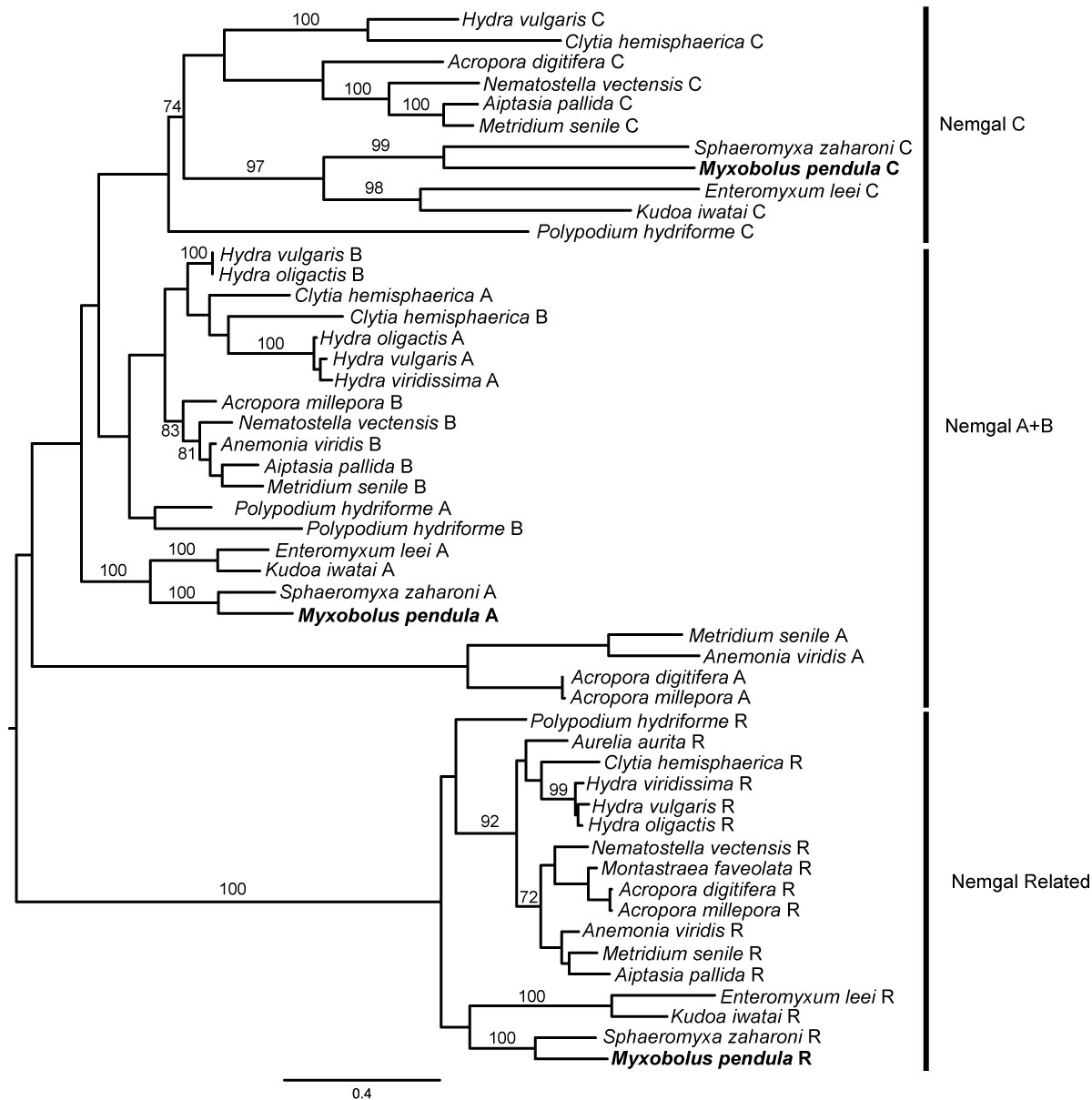

Supplement: Additional file 2: — Maximum likelihood phylogenetic reconstruction of nematogalectin family genes. Newly sequenced Myxobolus pendula nematogalectins are bolded. Numbers at nodes represent bootstrap support values. Values at nodes represent bootstrap supports ≥ 70. Nodes without values indicate bootstrap support below 70. (JPEG 175 kb) [file 12864_2015_2039_MOESM2_ESM.jpg]
