# Supplementary material for: In silico hybridization enables transcriptomic illumination of the nature and evolution of Myxozoa
Source: BMC Genomics. 2015 Oct 23;16:840. doi: 10.1186/s12864-015-2039-6 (PMC4619090; doi:10.1186/s12864-015-2039-6)
Supplement: Additional file 3: — Alignments of taxonomically restricted genes, including minicollagens (4) and nematogalectins (3). Taxa include previously sequenced myxozoans, newly sequenced Myxobolus pendula, and Polypodium hydriforme. Colored boxes refer to domain structure. Green = signal peptide; gray = propeptide; yellow = cysteine-rich; blue = polyproline (minicollagens), sugar-binding galectin (nematogalectins); red = polytripeptide; orange = tripeptide with alanine replacing glycine. Cysteine residues within cysteine-rich domains are shaded red. (DOCX 234 kb) [file 12864_2015_2039_MOESM3_ESM.docx]

**Supplementary Material 1.** Alignments of taxonomically restricted genes, including minicollagens (4) and nematogalectins (3). Taxa include previously sequenced myxozoans, newly sequenced *Myxobolus pendula*, and *Polypodium hydriforme.* Colored boxes refer to domain structure. Green = signal peptide; gray = propeptide; yellow = cysteine-rich; blue = polyproline (minicollagens), sugar-binding galectin (nematogalectins); red = polytripeptide; orange = tripeptide with alanine replacing glycine. Cysteine residues within cysteine-rich domains are shaded red.

Minicollagen 1 (Group 1)

Polypodium_hydriforme_Ncol1 --MLTRLSVPLMLLLGVAFAGIPRELEKRSPQ-YCDSGCPSYCAPSCQPICCI-------

Kudoa_iwatai_Ncol1 --MVMFTLPILVCLFSYTLASLPRSSEKRSPQAVCDYGCPAVCAPACLPVCCVA------

Enteromyxum_leei_Ncol1 ----MLTLSLLFCFISYTFASLPKSVEKRSPQ-LCDAACPAYCAPACTPICCA-------

Sphaeromyxa_zaharoni_Ncol1 ----MLSSLIASILIPFTISGLPRVKEKRQVYPYCPAPCPATCAPACLPVCCYSMAAVPA

Myxobolus_pendula_Ncol1 --MKTSQVLTFVFGISTVLAGLPRSIEKRQT--YCSPPCPTFCAPSCSPVCCYA------

Polypodium_hydriforme_Ncol1 ----------P-APPPPPPPPGPPGSPGNVGLPGPFGPPGPPGIPGIPGFPGQPGSPGVP

Kudoa_iwatai_Ncol1 ----------AAAPALPPPPPGPQGSPGPAGQPGPQGPPGPPGPPGPPGLPGGAGSPGQP

Enteromyxum_leei_Ncol1 ----------PAAPALPPPPPGPMGQSGQPGQPGPIGPPGPPGPPGPPGPSGSSGSPGYP

Sphaeromyxa_zaharoni_Ncol1 VAAIPAVAAVPALPPLPPPPPGPMGQPGPIGPPGPPGPPGLPGVQGMPGPLGSAGSPGYP

Myxobolus_pendula_Ncol1 ----------AAPPPLPPPPPGPMGVPGPSGPMGPPGPPGPPGSPGVQGSMGAAGSPGYP

Polypodium_hydriforme_Ncol1 GAPAGIPGVNGPQGPQGSAGPPGGPGLPGPPGPPGRPGSPGAPAPPPPPPPCPVVCTMQC

Kudoa_iwatai_Ncol1 GAAAGPPGPNGPAGPMGPRGNMGQPGLPGPPGPPGPPGLPGAPAPPPPPPPCPYVCTKTC

Enteromyxum_leei_Ncol1 GAAAGVP---------------GRPGLPGPPGPPGAPGAPGAPAPPPPPPPCPLMCTRKC

Sphaeromyxa_zaharoni_Ncol1 GAAAGIPGPNGAPGPLGAPGFMGPPGPPGPPGPPGPSGLPGAPAAPPPPPPCPYVCTTTC

Myxobolus_pendula_Ncol1 GAAAGAPGPNGPPGPSGQAGLMGQPGPQGPPGPPGPPGMPGAPAPPPPPPPCPYVCTTTC

Polypodium_hydriforme_Ncol1 TKTCHPTCCYKH

Kudoa_iwatai_Ncol1 TTSCHPTCCAKH

Enteromyxum_leei_Ncol1 VETCHPQCCFKH

Sphaeromyxa_zaharoni_Ncol1 LPTCHPTCC-KH

Myxobolus_pendula_Ncol1 LPTCHPTCCRR-

Minicollagen 2 (Group 2)

Polypodium_hydriforme_Ncol2 M-IHRSVVLLALVAVASCGL------PRNIEKRSPQSCDLGCQAVCAPTCLPICC---L

Buddenbrockia_plumatellae_Ncol2 M-INNELKIMRILTIISTLSC----LSTHYYVQRENSCQNXCPLRCYPSCLPNCC---S

Kudoa_iwatai_Ncol2 MGVGIDVTLLLVLIIYAHPSYT----KNPTKKQYINQCPPICATNCVPACPALCC----

Enteromyxum_leei_Ncol2 MWNTLLLLILSSHLYVTEP----ILSKYNEKKQRIAVCSPACESQCIPTCPAVCC---V

Sphaeromyxa_zaharoni_Ncol2 MFVTALVGINFLLISFSVPL------KEVLKRHIGISCPPLCQSYCYSYCPPTCCAAPL

Myxobolus_pendula_Ncol2 MLDFILTTISVLFYVASTDL--YSHSGDVAKKQYISFCPSQCASTCYPYCPAACCYGSY

Polypodium_hydriforme_Ncol2 PPPPPPPPGPPGSPGPVGLSGPSGPPGPPGAPGSPGLPGLPGPVGLPGAAAGSPGVNGP

Buddenbrockia_plumatellae_Ncol2 PIPPPPPPGPPGIPGPQGLTGPVGLPGLMGPPGQPGLAGQPGLPGNPGQQGPPLEI-CK

Kudoa_iwatai_Ncol2 SSLSPPPPGPVGSPGPPGLPGPQGPNGPPGPPGPPGPPGPAGSPGEPAPQAPPPQI-CP

Enteromyxum_leei_Ncol2 SNLPAPPPGPPGIPGQVGIPGQPGPNGPPGPVGPQGPPGPPGSPGMPAPPSPPVKM-CT

Sphaeromyxa_zaharoni_Ncol2 PPLPPPPPGPMGQPGPPGLAGPQGPPGPPGPPGRPGIPGFRGSPGLAGIPAPPPQV-CP

Myxobolus_pendula_Ncol2 PALPPPPPGPMGPPGPPGLTGPQGIPGTPGAQGPRGAPGPPGIPGQPGQPAPPPAV-CP

Polypodium_hydriforme_Ncol2 QGPQGGNGPQGP-------PGLPGPPGPP--GRPGL----------PGAPA-PPPP-P-Buddenbrockia_plumatellae_Ncol2 IECYQTCSDSCPKYCCSNSQTQP--------TCPDFCSQQCVPGVCPNSCCTNVPAELA

Kudoa_iwatai_Ncol2 LSCYTECVETCPQYCCVGPMPSPPPPPPPQIVCPPTCTVDVCAIDCPTECCVQPPP--P

Enteromyxum_leei_Ncol2 MECLTTCAPSCPTYCCPQEVVSTTPPPP---VCPPICTVTTCISSCPSDCC-QPPA--P

Sphaeromyxa_zaharoni_Ncol2 VSCYTVCAPTCPTYCCAAP---PPPPPPP--VCPAICE-TTCAPICPPVCC-LPPT--P

Myxobolus_pendula_Ncol2 TTCYSICAPSCPSYCCSEQPAPPPPSPPPPVVCPTICS---------------------

Polypodium_hydriforme_Ncol2 PP----CPVVCTVQC-TRTCHPTCCAKH-----------------

Buddenbrockia_plumatellae_Ncol2 QVQTQPCPEICQTQCIKPLCSTSCCSPYFKRTLNDDHDXENNXFI

Kudoa_iwatai_Ncol2 PPTSLVCPPICQVSC-APVCPTECCTKHRRHHILSTKEKSMD---

Enteromyxum_leei_Ncol2 TPSTSNCPAICQATC-APICPSSCCKKRKRHHILSSQAQYID---

Sphaeromyxa_zaharoni_Ncol2 PP--VACPPVCSTTC-APVCPPICCAKHKRQNILSKENIQQEN--

Myxobolus_pendula_Ncol2 ----TSCTSIC--------------QIGR-A--------------

Minicollagen 3 (Group 2)

Polypodium_hydriforme_Ncol11 ----MAMFLPLLLLVWVGAEAKSLHEML-----RR--EANPCGSACPSYCAPSCLTSCCA

Buddenbrockia_plumatellae_Ncol3 ---MKLILGILLLTYLIDVYGEKSLF-------RR--QVNTCSPGCPTSCYPECTPTCCA

Kudoa_iwatai_Ncol3 MIRGVFLLLSTVALSFAATEAEKVY--------KRSPQVNVCGPVCPPICAPACTVQCCT

Enteromyxum_leei_Ncol3 -MFKEVSGLVLFLSTIALVRADKGDKVF-----KRSPQYDSCGPACPPTCAPSCSVQCCA

Sphaeromyxa_zaharoni_Ncol3 ----MNHLSLLLVSAIVVVHSKNIDGVF-----KR--SSYPCGYPCPISCAPACLPACCA

Myxobolus_pendula_Ncol3 --MVAIFGIISAVLVGAAAKSIDGTYKLYLAAVKR--DLYPCGNTCPSYCAPACSPVCCA

Polypodium_hydriforme_Ncol11 --------------------------------------------PAGPIPALPGPPGPPG

Buddenbrockia_plumatellae_Ncol3 --------------------PQQQVYYPP-------PPPP---PPSPPIPALPGPPGPPG

Kudoa_iwatai_Ncol3 AP----------------PPPPPPIYIPPPP-----PPPPPPPPPPPPLPALPGPPGPPG

Enteromyxum_leei_Ncol3 PP----------------PPPPPPPPPPPPPPPVYYPPPPPPSPPPPPLPALPGPPGPPG

Sphaeromyxa_zaharoni_Ncol3 APAPAPVYVAPAPAPVYVPPPAPPVYVPP-------PPPPPPLPPLPPLPALPGPPGMPG

Myxobolus_pendula_Ncol3 ------------------PPPPPPVYIPP-------PPPPPPPPPPPPIQALPGPPGPPG

Polypodium_hydriforme_Ncol11 RPGPPGPMGMPGMPGPPGPPGAPGASGSPGTPGAPAPPPAPCPSSCQSQCVSSCPMYCCP

Buddenbrockia_plumatellae_Ncol3 RPGAMGPMGPPGMQGPMGPPGQQGSPGSPGIPGSPAPPPKPCQPSCATNCIMACPQYCCP

Kudoa_iwatai_Ncol3 KPGPAGLMGPPGPQGPPGPPGPPGISGTPGAPGAPAPPPAPCPVFCQTRCVDSCPLYCCP

Enteromyxum_leei_Ncol3 KPGSAGLMGPPGVAGPPGPPGPPGVSGTPGAPGAPAPPPVQCPSSCITQCTQSCPMYCCP

Sphaeromyxa_zaharoni_Ncol3 KPGPSGLMGPPGPPGAPGAPGAAGQPGVPGQPGAPAPPPAPCPPICATQCVMDCPLYCCP

Myxobolus_pendula_Ncol3 KPGPSGLMGPPGPPGPPGQAGAPGMAGNPGQPGSPAPPPAPCPPVCQTQCVMDCPLYCCP

Polypodium_hydriforme_Ncol11 ARK-

Buddenbrockia_plumatellae_Ncol3 VV--

Kudoa_iwatai_Ncol3 ARR-

Enteromyxum_leei_Ncol3 ARRR

Sphaeromyxa_zaharoni_Ncol3 TKK-

Myxobolus_pendula_Ncol3 SKK-

Minicollagen 4 (Group 3)

Polypodium_hydriforme_Ncol7 ---MMSYGWVLIGLVAVTSAMSLD-KRSAEPCDGAGCGG-CGD-C-----YGAGGYG--

Polypodium_hydriforme_Ncol8 -MSLIFAFSLAVVAVSGVWSAALE-KREAEPC-GYGCPSYCAPSCSSSCCGAGAGGAAY

Polypodium_hydriforme_Ncol9 --MCTFVVLSVLVLVSEMSAMTLD-KRSADAC-GYGCSPSCAPSCNPQCCSYMINPPPV

Myxobolus_pendula_Ncol4 MFLLGKIILIYNIFITHQGIINVKSKRSPQMC-GMGCPPMCAPSCNAMCCGMGGSGAAQ

Polypodium_hydriforme_Ncol7 -----APSYGVGG—YGGCPPSCASGPG-MIAA------GPQGSPGAMGFPGPMGPPGAP

Polypodium_hydriforme_Ncol8 YPAP-APACGYAS--APACAPAAAAAPMM-------IPGPPGAPGMMGSPGFMGPAGAP

Polypodium_hydriforme_Ncol9 PPPPMAPTCMYPSSCYAPAPPACMASP-MCASLPASIPGPPGPPGCMGPMGSPGCAGLM

Myxobolus_pendula_Ncol4 PPPPPPPPP--------------------TAIL---IPGPPGPPGPPGMGASGGGLGGN

Polypodium_hydriforme_Ncol7 GFMGPPGPMGPPGVPGFPGVPGAPGASCPPICITHCMRICPLSCC---------TASPL

Polypodium_hydriforme_Ncol8 GMMGAPGPMGPPGSPGMPGAPGAPGASCPPICVTHCMRICPLPCC-------------A

Polypodium_hydriforme_Ncol9 GAPGMPGAPGMPGAPGVPGAPGVPGASCPPICIQHCMRICPMSCC-------------A

Myxobolus_pendula_Ncol4 ---------------------------CPPICITTCIRGCPPQCCLPGGGAGGMGGG-A

Polypodium_hydriforme_Ncol7 PPPPPP----M-CMPAPCSPP--------------------SY----------------

Polypodium_hydriforme_Ncol8 PPPPPPPP-QMACAMPSCMPPPPPPM------CMPQPCSPPSP----------------

Polypodium_hydriforme_Ncol9 PPPPPPPP--V-CMPAPCAPPPP---------CMAPPCAMQTP----------------

Myxobolus_pendula_Ncol4 PPPPPPPPPQVICLPPMCAPPPPPPPAGSQIICLPPQCMPQPPPMPSPMICPPCPASAR

Polypodium_hydriforme_Ncol7 ----------CCG

Polypodium_hydriforme_Ncol8 ----------CCG

Polypodium_hydriforme_Ncol9 ----------CCG

Myxobolus_pendula_Ncol4 PPPMCPPAMGCC-

Nematogalectin A

Polypodium_hydriforme ------------MWWPTLSLLL—FCLLDNHESEGQRM MPEWPHVGDRVSQSFLDQLMVS

Enteromyxum_leei -----MHKYNAEFQWNFFKFFL--ISYITIVSSENFHPQLNLPQIGDVVTEEMINQLMIS

Kudoa_iwatai MADQIKNAINLCSLFSTYTYYL--IILGVVMSQR--PPQLNLPQLGDVVDQNLIDQIMIS

Sphaeromyxa_zaharoni ------------MILINIKPYIIGIFFILEIKHNQCQRQIVLPQVGEPITQQMIDQLMIS

Myxobolus_pendula ---------MQKIFCINIFFVL-LKIQSCEKQI-------VLPSLGEPVTQQMIEQIMIS

Polypodium_hydriforme QLLEQNLTLGFFLKGLNGPPGPPGPTGPAGDPGPPGMPGAPGLPGHVGEDGAPGPIGLQG

Enteromyxum_leei NLIAQNMTVGFFLRGLNGPPGPPGPPGEAGQPGEPGSQGPPGLQGQVGEDGAPGPKGPSG

Kudoa_iwatai NLVSQNLTMGFFLRGLNGPPGPPGPPGEPGVPGEPGLPGAPGLQGQVGEDGAPGPPGPRG

Sphaeromyxa_zaharoni QLLSQNLTMGFFLRGLNGPPGEPGMPGAPGDPGPPGFPGAPGLPGSVGEDGAPGPAGPTG

Myxobolus_pendula QLIAQNLTMGFFLRGINGMPGPPGLPGPSGPPGNPGYPGPPGLPGQIGEDGAPGPQGPPG

Polypodium_hydriforme PPGMAGPPGPPGSKGDTGMSGIPGEPGLPGAPGLPGLPGPMGPPGSDPLMPNFTVICEGE

Enteromyxum_leei EIGAPGAPGLTGPKGDPGEQGIPGAQGPKGDPGDIGPPGMPGSVGSDLISPNYTVICEGE

Kudoa_iwatai EMGPPGAPGLTGAKGDPGEQGIPGAQGIPGEPGPMGPPGLPGSSTGDLITPNYTVICEGE

Sphaeromyxa_zaharoni NTGAPGAPGLRGPQGEPGEQGSPGPPGPPGHPGPVGPPGEPGSPAPEIFMPNYTVICEGE

Myxobolus_pendula QPGSPGAPGITGPKGDSGEQGIPGPPGEPGPPGPVGPIGPPGAPAPEMLLPDYTVLCEGE

Polypodium_hydriforme KGWLQCKQYELVKVTRAFWGRDDYSTCPNAPAGLTTERLCETGAENTLAKVNNQCKNSQA

Enteromyxum_leei KGWIQCKQYEVVNIIKVFWGRDDFSTCEKAPAGLTTERLCETNSDDAFTKINDQCKNTQA

Kudoa_iwatai KGWIQCKQYEVVNVIKVFWGRDDFTTCEKSPAGLTTDRLCETNTDDALAKINDQCKNTQA

Sphaeromyxa_zaharoni KAWIQCKQYEVVTINKVYWGRDDYTTCDKVPAGLTKDRLCDANEEEAYEKVVDQCRNKQA

Myxobolus_pendula KALIQCKQYEVVSINKVYWGRDDYTTCDKVPANLTKDRLCDTNQKEAFEKVTDQCQNKQA

Polypodium_hydriforme CEVVASNIFFDDNSCGNVFKYLKLWYECIADEANAVDVLRDGNRKKRRQATKDKRNLRDE

Enteromyxum_leei CEVVATNLFFNDNTCGNVYKYLKLW-----------------------------------

Kudoa_iwatai CEVVATNLFFNDNSCGNVYKYLKLWYDCVPDEVNAVDVLRDEARKRRRSVKAKRHTVV--

Sphaeromyxa_zaharoni CEVVATNIFFNDNSCGNVYKFLKIWYDCMPDDLNSIDVPKDGEKRRKRWIIVEN------

Myxobolus_pendula CEVVATNIFFNDNSCGNVYKFLKIWYDCKPDDLNAVDMGKDGLKRRKRFSILDRNL----

Nematogalctin-related

Polypodium_hydriforme MTLPAMSFR--RMVPKW-AWLVHSILLIVIFAQPAVSVPQ-FQVPPLLNQLLRDQNVTLG

Enteromyxum_leei MVFWYYSETSSKIIWK----LC--IIIYLSF-LYRINAQN-SSIPPLLNQLLKDQKVTLG

Kudoa_iwatai MIFHYISTNTTKKIPI----ILFIYILLMNM-LHKVAVQRPGQIPPLLNQLLKDQNVTLG

Sphaeromyxa_zaharoni MNIIAP-----RKIFKFKFFVCCLIIFYVNF--KVVSNQS-LPIPPLLDQLLRDQNVTLG

Myxobolus_pendula ML---------NHLNKY-YFICFTYFLFLNF-KNLKALPN-LPVPPLLNQLLHDQNVTLG

Polypodium_hydriforme FILKGLQGPPGKDGLPGMPGQPGLMGPQGMPGDPGGPGAPGLMGPMGPPGLQGNPGQDGW

Enteromyxum_leei FILKGLQGPPGMDGMQGPPGMIGPGGPPGMTGEMGPMGPPGMRGFTGEPGVAGEPGRDGL

Kudoa_iwatai FILKGLQGPAGFDGIPGAPGVQGPIGPPGYPGEMGPMGPPGLRGFPGEPGVPGEPGRDGN

Sphaeromyxa_zaharoni FILKGLQGPPGMDGSPGYPGPPGLPGPIGFTGEMGPMGPPGSKGDKGETGYPGKPGMDGW

Myxobolus_pendula FILKGLQGPRGLDGSPGYPGPPGLPGPIGYPGDIGPLGPPGPQGPKGDLGAPGRPGIDGW

Polypodium_hydriforme PGAPGAPGMTGAPGSSGMPGPPGLPGLQGAPGAPGPTAIRY-NGTVKCEEDTAWLRCGEY

Enteromyxum_leei DGIQGPPGIPGDPGPAGMSGPPGPPGTPGTINGVVETRFNIPNITLKCEEDTAWLKCGEY

Kudoa_iwatai DGYPGAPGFPGEPGPSGMPGPPGPPGLPGDTPPFLPIVLRN-TTIIKCEEDTAWLKCVDY

Sphaeromyxa_zaharoni IGPPGSPGFPGEPGNSGPEGQAGPPGIPGEPGPPGLSSIRY-NGTVKCEEDTAWLRCGEF

Myxobolus_pendula IGPPGPAGFPGTPGSSGPPGNPGSPGMPGEMGPPGLSAIKY-NGTVKCEEDTAWLRCTEF

Polypodium_hydriforme KRISIISAFWGRRNFALCTEHTGNLNSKKYCPTQPLFLTKVKDACEGTTICEIRCTKFFF

Enteromyxum_leei KRISVKSVFWGRRDFEKCAENNGNLFVDKYCPTQPLFLAKVKDACEGTTMCEIRCTKLFF

Kudoa_iwatai KKISIKSVFWGRRNFDICSENSGNLVTDKYCPTNPLFLAKVKDACDGTTMCEIRCTKLFF

Sphaeromyxa_zaharoni KRISIVSVFWGRRSLAVCAEHTGDLYTDKFCPTDPMFLTKVKDTCEGTTMCEIRCTKTFF

Myxobolus_pendula KRINIISAFWGRRDMGMCAEHTGDLKTDIYCPTQPIFLTKIKDTCEGTTICEIRCTKRFF

Polypodium_hydriforme HDKTCPDVYKYLEVYYKCIEVINGHEVVNEDNLLSANFAG

Enteromyxum_leei NDKSCPDVYKYAEIDYDCVEIINGHEVVNNRHIIVE----

Kudoa_iwatai NDKTCPDVYKYAEIDYKCVEVINGHEVVNNERNVMGEI--

Sphaeromyxa_zaharoni NDNHCPEIYKYLEVYYKCIEVINGHEVVNEENVLSGNMFG

Myxobolus_pendula NDNTCPEVYKYLEIFYKCIEVINGHEVVNEENLLSANMFG
